# Loss of Atg16 delays the alcohol-induced sedation response via regulation of Corazonin neuropeptide production in Drosophila

**DOI:** 10.1038/srep34641

**Published:** 2016-10-06

**Authors:** Kata Varga, Péter Nagy, Katarina Arsikin Csordás, Attila L. Kovács, Krisztina Hegedűs, Gábor Juhász

**Affiliations:** 1 Department of Anatomy, Cell and Developmental Biology, Eötvös Loránd University, Pázmány s. 1/C, Budapest, H-1117 Hungary; 2 Institute of Genetics, Biological Research Centre, Hungarian Academy of Sciences, Temesvári krt. 62, Szeged, H-6726 Hungary

## Abstract

Autophagy defects lead to the buildup of damaged proteins and organelles, reduced survival during starvation and infections, hypersensitivity to stress and toxic substances, and progressive neurodegeneration. Here we show that, surprisingly, Drosophila mutants lacking the core autophagy gene Atg16 are not only defective in autophagy but also exhibit increased resistance to the sedative effects of ethanol, unlike Atg7 or Atg3 null mutant flies. This mutant phenotype is rescued by the re-expression of Atg16 in Corazonin (Crz)-producing neurosecretory cells that are known to promote the sedation response during ethanol exposure, and RNAi knockdown of Atg16 specifically in these cells also delays the onset of ethanol-induced coma. We find that Atg16 and Crz colocalize within these neurosecretory cells, and both Crz protein and mRNA levels are decreased in Atg16 mutant flies. Thus, Atg16 promotes Crz production to ensure a proper organismal sedation response to ethanol.

The catabolic process of autophagy ensures cellular and organismal homeodynamics via the lysosomal degradation and recycling of intracellular material. Atg gene products promote the formation of autophagosomes: double-membrane vesicles that transport cytoplasmic cargo to lysosomes. Upregulation of autophagy ensures the survival of cells and organisms during nutrient limitation, hypoxia or other adverse conditions. In addition, low-level basal autophagy contributes to the turnover of biological macromolecules including proteins, lipids, RNA, and even whole organelles such as mitochondria or ER. This housekeeping function of autophagy is thought to be especially important in long-lived, terminally differentiated cells such as neurons, because the neuron-specific loss of Atg5 or Atg7 leads to the accumulation of ubiquitinated protein aggregates and neurodegeneration^1^,^2^. Similar phenotypes can be detected in Drosophila mutants lacking Atg7 or Atg8a, indicating that autophagy plays an evolutionarily conserved role in maintaining neurons^3^,^4^.

Loss of autophagy leads to a range of phenotypes in Drosophila that include delayed development, climbing defects due to neuromuscular dysfunction, increased sensitivity to starvation and oxidative stress, memory defects and short lifespan^3^,^4^,^5^. One could argue that loss of autophagy decreases the overall fitness of animals, which manifests in hypersensitivity to various toxins and stressors, and poor performance in these tests. Interestingly, the severity of various Atg mutant phenotypes differs considerably in Drosophila. Mutation of genes encoding subunits of the upstream acting Atg1 and Vps34 kinase complexes results in highly penetrant lethality during metamorphosis^6^,^7^. In contrast, the deletion of genes encoding proteins involved in the ubiquitin-like protein conjugation systems so far proved to be viable. These include the ubiquitin-like protein Atg8a, and Atg7, the E1-like enzyme that is required for the C-terminal lipidation and autophagosomal localization of Atg8a, together with the E2-like Atg3^3^,^8^. Atg7 also activates Atg12, another ubiquitin-like protein, which then becomes covalently bound to Atg5. The Atg5-12 conjugate then forms a large protein complex together with Atg16, and this complex is thought to function similar to an E3 enzyme by promoting the final step of Atg8a lipidation^9^. Of note, we have recently shown that Atg5 knockout flies are also viable^10^.

In this work we generated null mutants for Drosophila Atg16. These animals exhibit the expected autophagy defects and as a consequence, the accumulation of neuronal protein aggregates, climbing defects, sensitivity to the oxidative stress-inducing toxin paraquat, and short lifespan. Surprisingly, however, we find that the loss of Atg16, but not Atg7 or Atg3, leads to greatly increased resistance to the sedative effects of ethanol in adult flies. We show that Atg16 function in ethanol sensitivity maps to Corazonin-producing neurosecretory cells located in the pars lateralis using cell type-specific genetic rescue and RNAi experiments, and that Atg16 deficiency impairs Corazonin production. These data reveal an unexpected new role for Atg16 in this popular animal model.

## Results

### Generation and characterization of Atg16 mutants

**Figure 1:**
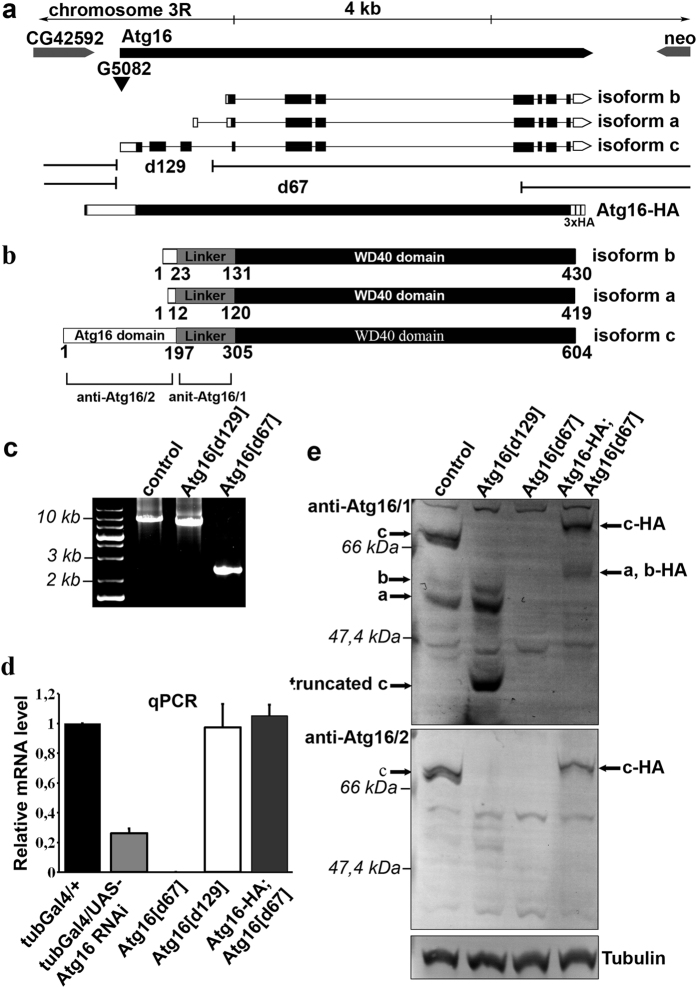
Generation of Atg16 mutations, rescue transgene, and antibodies. (**a**) Two Atg16 mutations were isolated by improper excision of the P element G5082, which is located in the 5′ UTR of this gene. Atg16^d129^ carries a 1,977 bp deletion removing the first part of the Atg16 gene, and Atg16^d67^ contains a 7,914 bp deletion removing much of the gene. The composition of the genomic promoter-driven Atg16-HA transgene is also shown. The Atg16 mRNA isoforms are indicated, with open bars representing untranslated regions and closed bars representing coding sequences. (**b**) This panel illustrates the domain structures of Atg16 protein isoforms, and the recognition regions of anti-Atg16/1 and 2. (**c**) PCR amplification from genomic DNA with primers specific for flanking regions of the Atg16 gene shows the size of the deletions in Atg16^d129^ and Atg16^d67^ adult flies compared to control. (**d**) Quantitative real-time PCR shows a decrease of Atg16 mRNA level in Atg16 RNAi and Atg16^d67^ mutant flies compared to control and rescued Atg16^d67^ mutants. No decrease of mRNA expression is seen in Atg16^d129^ mutants because primers are specific for a common region of the 3 mRNA isoforms. Standard error values are ±0.031131; 0.000001; 0.0156954; 0.052920 (the tub-Gal4/ + control was set to 1). (**e**) Western blots using linker region-specific (anti-Atg16/1) and Atg16 domain-specific (anti-Atg16/2) antibodies show the expression of the various Atg16 isoforms in control, Atg16^d129^ and Atg16^d67^ mutants, and in Atg16^d67^ mutants rescued by Atg16-HA.

Atg16 is predicted to encode 3 mRNA and protein isoforms in Drosophila, based on high-throughput RNAseq data^11^. Isoform c contains the N-terminal Atg16 domain that is necessary for autophagy through its binding to Atg5, a central linker region, and a C-terminal WD40 domain. Isoforms a and b are shorter because they lack the N-terminal Atg16 domain (Fig. 1a,b).

We generated two deletions by imprecise excision of the P element G5082 located in the 5′ non-coding region of Atg16 isoform c (Fig. 1a–c). The Atg16^d67^ allele removes the first 6 exons and part of the 7th exon of isoform c. As this Atg16^d67^ allele affects 4 of the common exons shared by all 3 isoforms and deletes the transcriptional start sites of isoforms a and b, this likely creates a null mutant. The Atg16^d129^ allele removes the first 3 exons of isoform c only. Both mutants are homozygous viable and fertile and are maintained as a stable stock, and showed no obvious morphological defects.

Quantitative real-time PCR (qPCR) experiments detecting a shared region of the 3 mRNA isoforms showed the lack of gene expression in homozygous Atg16^d67^ animals, whereas mRNA levels were similar in control animals, Atg16^d129^ mutants, and Atg16^d67^ animals rescued with a C-terminally 3xHA tagged Atg16 expressed from its own promoter (Fig. 1d). In addition, RNAi knockdown of Atg16 resulted in a 75% reduction of mRNA levels (Fig. 1d).

We also characterized Atg16 protein expression in wild type and mutant animals using two anti-Atg16 antibodies that we generated. The first one (anti-Atg16/1) was produced against the common linker region found in all 3 isoforms, while the second one (anti-Atg16/2) recognizes the Atg16 domain found only in the longest isoform c (as shown in Fig. 1b). Western blots using these antibodies confirmed that Atg16^d67^ is a null allele lacking all protein isoforms, and we detected a new protein band in addition to isoforms a and b in Atg16^d129^ mutants (Fig. 1e). This probably represents an N-terminally truncated form of isoform c lacking the Atg16 domain and part of the linker sequence, as a likely result of utilizing an alternative translational start site in its truncated mRNA. As expected, expression of the C-terminally 3xHA tagged Atg16 restored Atg16 protein expression in Atg16^d67^ mutants (Fig. 1e).

### Drosophila Atg16 is required for autophagy in fat cells and neurons

**Figure 2:**
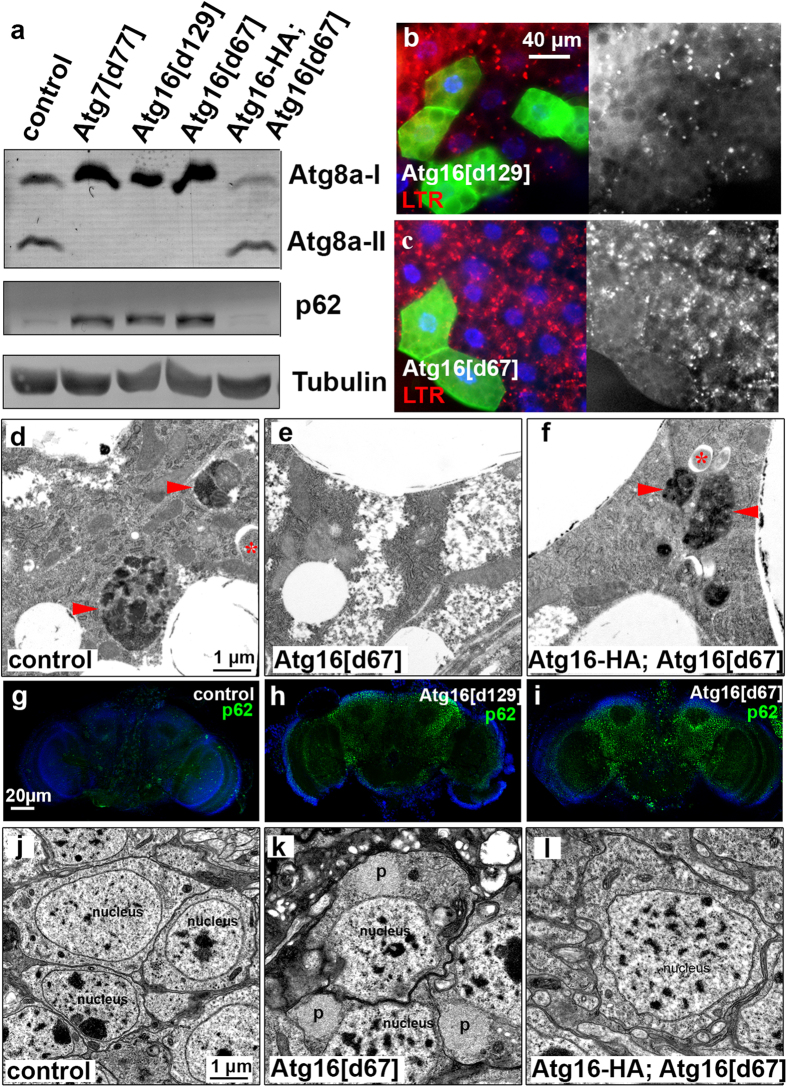
Atg16 is required for autophagy in larval fat and adult brain cells. (**a**) Lipidated, autophagosome-associated Atg8a (Atg8a-II) is missing and the selective autophagy cargo p62 accumulates in Atg16^d129^ and Atg16^d67^ mutant larval lysates, similar to Atg7^d77^ mutants. These phenotypes are rescued by Atg16-HA expression in Atg16^d67^ mutants. (**b**,**c**) Starvation-induced punctate LTR staining is blocked in homozygous Atg16^d129^ (**b**) and Atg16^d67^ (**c**) mutant fat body cells that are marked by GFP expression, compared to neighboring GFP-negative control cells. (**d**–**f**) Ultrastructural images of fat cells in starved L3 larvae. Autophagosomes (asterisks) and autolysosomes (arrowheads) are seen in control (**d**) and genetically rescued (**f**) animals, unlike in Atg16^d67^ mutant fat body cells (**e**). (**g**–**i**) Endogenous p62-positive protein aggregates (green dots) accumulate throughout the brain of Atg16^d129^ (**h**) and Atg16^d67^ (**i**) animals compared to control brain (**g**). (**j**–**l**) Ultrastructural analysis of 20-day-old brains reveals the presence of large protein aggregates (p) in Atg16^d67^ mutant neurons (**k**), which are absent from control (**j**) or genetically rescued (**l**) animals.

Atg16 is important for the lipidation of Atg8 family proteins^9^, so we next investigated Atg8a modification status in our mutants. Western blots revealed that Atg8a lipidation was severely impaired in both Atg16^d67^ and Atg16^d129^ mutant larvae (Fig. 2a). In line with this, we found large-scale accumulation of p62 (also known as Ref(2)P in flies), the selective autophagy cargo and ubiquitinated protein receptor^8^,^12^, in both Atg16^d67^ and Atg16^d129^ mutants compared to controls. Restoration of Atg16 expression fully rescued these autophagy defects in Atg16^d67^ mutant larvae (Fig. 2a).

We further confirmed the inhibition of autophagy in Atg16^d67^ and Atg16^d129^ homozygous mutants using microscopy. Starvation induces the formation of acidic autolysosomes in larval fat cells, which can be detected by staining with Lysotracker Red (LTR)^13^,^14^. This starvation-induced Lysotracker staining was blocked in GFP-positive fat cell clones that are homozygous mutant for either Atg16^d129^ or Atg16^d67^, compared to surrounding GFP-negative control cells in mosaic animals (Fig. 2b,c). Transmission electron microscopy also showed that starvation-induced autophagosome and autolysosome generation is impaired in fat cells of Atg16^d67^ mutant larvae, unlike in controls or genetically rescued mutants (Fig. 2d–f). Lastly, we estimated autophagic flux based on the level of p62 in the adult brain^13^,^14^. As expected, both Atg16^d67^ and Atg16^d129^ mutant adult flies accumulated large amounts of p62 aggregates relative to control brains (Fig. 2g–i). In line with this, ultrastructural analysis revealed the presence of protein aggregates in neurons of Atg16^d67^ mutant adult flies, which were absent in control or genetically rescued animals (Fig. 2j–l). This is similar to what we have previously observed in Atg7 and Atg8a null mutant flies^3^,^13^.

### Physiological consequences of Atg16 deficiency

**Figure 3:**
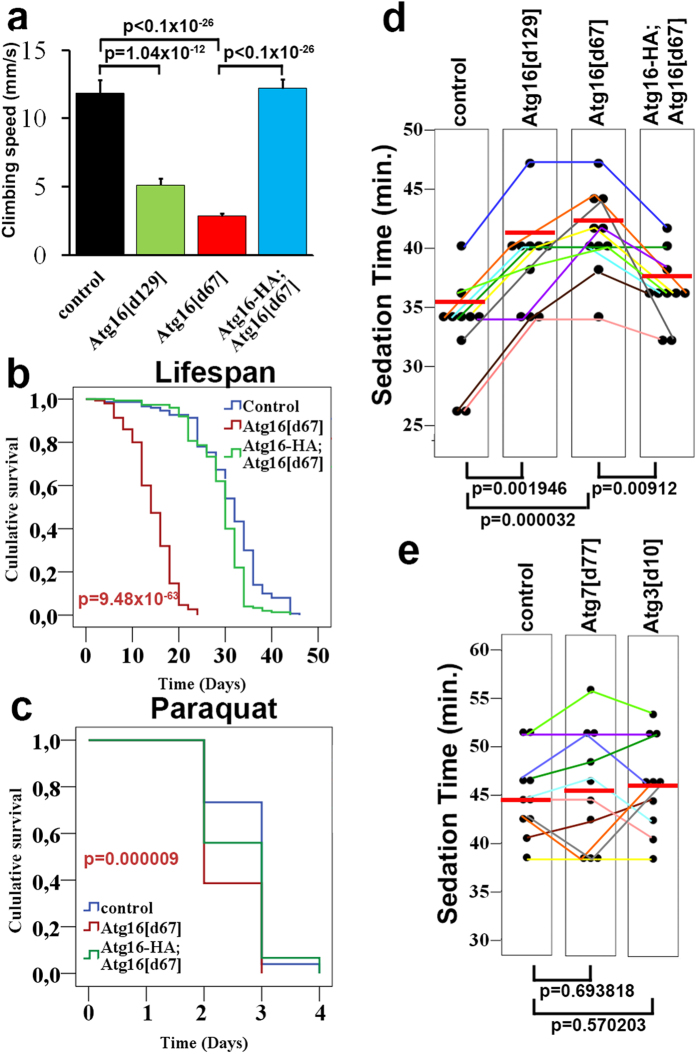
Physiological effects of Atg16 mutations. (**a**) Atg16^d129^ and Atg16^d67^ male flies show reduced climbing ability in negative geotaxis assays compared to control or genetically rescued Atg16^d67^ flies. Standard error values are ±0.960136; 0.465029; 0.213002; 0.647568. (**b**) Homozygous Atg16^d67^ mutant flies have a shorter lifespan when compared to control or genetically rescued animals. (**c**) Atg16^d67^ mutant flies die earlier than control or genetically rescued animals in response to treatment with the toxin paraquat. (**d**) Quantification of ethanol sedation responses of Atg16 mutant strains. Sedation Time 50 is the time required for sedating half of the flies in a vial. A statistically significant increase is seen in the case of Atg16^d129^ and Atg16^d67^ mutants compared to controls or genetically rescued (Atg16-HA; Atg16^d67^) flies, n = 10. Each dot represents a single data point, and the red line indicates the median in this and subsequent scatter plots. Data points from the same experiment are linked together by colored lines. Standard error values are ±1.384036; 1.36178; 1.127928; 0.879394. (**e**) The Atg7^d77^ and Atg3^10^ homozygous mutations do not affect ethanol sedation sensitivity in adult flies, n = 10. Standard error values are ±1.3888444; 1.868154; 1.50148075.

Loss of autophagy in the central nervous system leads to early onset neurodegeneration both in mice and flies, which manifests in neuromuscular dysfunction^1^,^2^,^3^. Using a well-established functional assay, we measured the climbing ability of our Atg16 mutant flies^3^,^10^,^13^. It is based on the negative geotaxis reflex of adult flies: when they are gently tapped down in a vial they immediately start climbing up, and their upward movement can be easily quantified. Both Atg16^d67^ and Atg16^d129^ mutants performed poorly in these negative geotaxis tests compared to control or genetically rescued Atg16^d67^ flies (Fig. 3a). Moreover, the lifespan of Atg16^d67^ mutant adult flies was much shorter than that of control or genetically rescued animals (Fig. 3b), and they were more sensitive to treatment with the oxidative stress-inducing toxin paraquat (Fig. 3c). These phenotypes are similar to what we found earlier in the case of Atg7 null mutants^3^.

The p53-induced gene Ei24 was recently identified to be essential for basal autophagy in both worms and mice^15^,^16^. The Drosophila homolog of this gene is called tank, because its mutation allows flies to inhale high amounts of ethanol vapor before becoming sedated^17^. Although the mechanisms of how tank/Ei24 regulates basal autophagy and ethanol sensitivity are not known, we were wondering whether Atg16 has a role in alcohol resistance.

In Drosophila, acute exposure of adult flies to ethanol vapor leads to a sedation response, which is characterized by loss of postural control and righting reflex^18^,^19^. To determine the ethanol sedation sensitivity of our Atg16 mutants, we counted the number of non-sedated versus sedated flies during the course of ethanol treatment. Both Atg16^d67^ and Atg16^d129^ mutant flies were much more resistant to sedation than control flies or Atg16^d67^ mutants rescued with the genomic Atg16-HA transgene (Fig. 3d). In contrast, the ethanol response of Atg7 and Atg3 null mutant adult flies was similar to controls (Fig. 3e), suggesting that Atg16 regulates alcohol sedation independent of these other Atg proteins.

### Atg16 expression in Corazinin-producing neurosecretory cells promotes ethanol sedation

**Figure 4:**
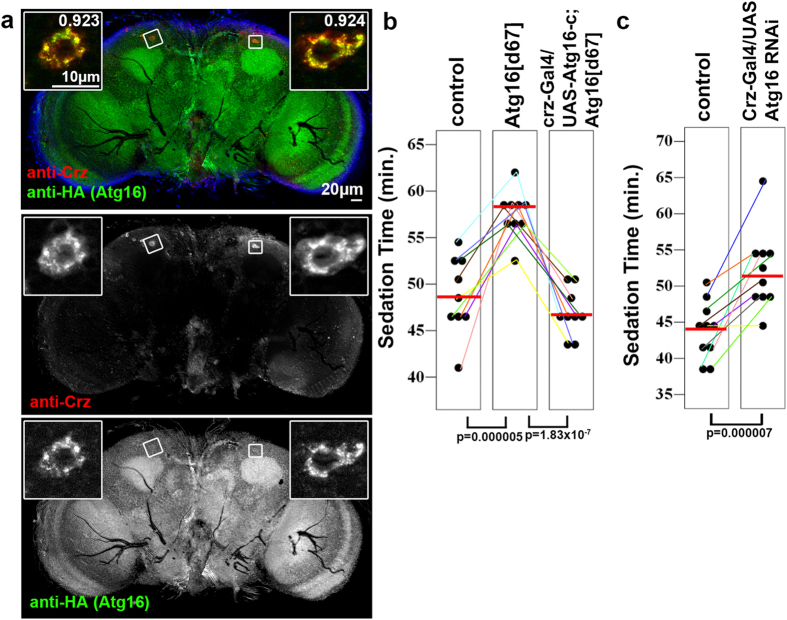
Atg16 functions in Crz-producing neurosecretory cells during the ethanol sedation response. (**a**) Atg16-HA is highly expressed in cells positive for the neuropeptide Crz, and the Atg16-HA and endogenous Crz signals largely overlap. Numbers in insets of the upper panel represent Pearson correlation coefficients, which indicate a near-complete colocalization of punctate HA and Crz signal in these cells. (**b**) The delayed ethanol sedation response of Atg16^d67^ mutants is completely reverted by re-expressing Atg16-c in Crz-positive neurons, n = 9. Standard error values are ±1.327534; 0.833333; 0.824022. (**c**) RNAi knockdown of Atg16 specifically in Crz-producing cells delays ethanol-induced coma, n = 10. Standard error values are ±1.366667; 1.678624.

Ethanol sedation is mediated at least in part by two groups of altogether 14 neurosecretory cells located in the pars lateralis of the fly brain, which produce the neuropeptide Corazonin (Crz)^19^. Crz is thought to be the homolog of mammalian gonadotropin-releasing hormone, the production of which is directly controlled by stress hormones. The expression of the genomic promoter-driven Atg16-HA transgene was elevated in Crz-positive cells compared to surrounding cells in the adult brain, and interestingly, the endogenous Crz and Atg16-HA signals largely overlapped within these cell bodies (Fig. 4a). We next carried out genetic rescue experiments by expressing the longest c isoform of Atg16 specifically in Crz-positive cells using a crz-Gal4 transgene to drive Gal4-dependent UAS-Atg16-c expression. Normal ethanol sedation sensitivity could be restored in Atg16^d67^ mutants by targeted expression of Atg16-c only in these neurosecretory cells, indicating that Atg16 function is specifically required in Crz-positive cells (Fig. 4b). In line with this, we also observed a delay of ethanol-induced sedation response when we knocked down Atg16 specifically in Crz-producing cells using the crz-Gal4 driver and the UAS-Atg16 RNAi transgene (Fig. 4c), which effectively inhibits Atg16 expression (Fig. 1d).

### Atg16 regulates Crz expression

**Figure 5:**
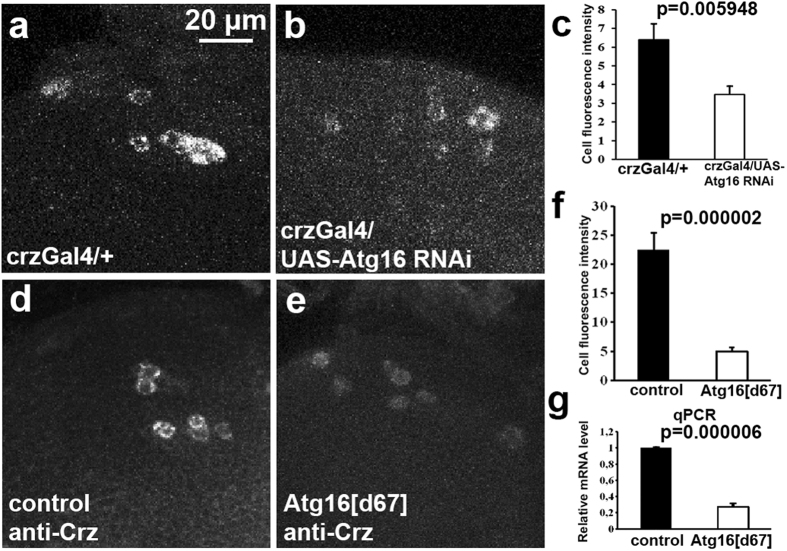
Loss of Atg16 decreases Crz protein and mRNA levels. (**a–c**) Expression of the genomic promoter-driven Crz-mCherry fusion protein (shown in grayscale) is reduced in Crz-producing cells undergoing Atg16 RNAi (**b**) compared to its level in control brains (**a**). (**c**) Quantification of data shown in (**a**,**b**), n = 5 brains/genotype. Standard error values are ±0.822780; 0.444095. (**d–f**) Endogenous Crz protein level (shown in grayscale) is decreased in Atg16^d67^ mutant neurons (**e**) compared to control brain (**d**). (**f**) Quantification of data shown in (**d**,**e**), n = 5 brains/genotype. Standard error values are ±2.950328; 0.756994. (**g**) Crz mRNA level is decreased in Atg16^d67^ mutant males compared to control animals. Standard error value is ±0.026742 (the control was set to 1). Note that all microscopy and qPCR analyses were done using male flies treated with ethanol vapor for 30 min.

Based on these results, we were wondering whether the loss of Atg16 could influence Crz production. Indeed, RNAi knockdown of Atg16 in Crz-producing cells strongly reduced the expression of endogenous promoter-driven, mCherry-tagged Crz (Fig. 5a–c), a new reporter line that we have generated. Similarly, we detected a clear reduction of endogenous Crz protein levels in Atg16^d67^ mutant adult flies (Fig. 5d–f). We next carried out qPCR experiments to further investigate the effect of Atg16 on crz gene expression. The analysis revealed that crz mRNA level is reduced to 25% in Atg16^d67^ mutants. We thus conclude that the loss of Atg16 increases resistance to the sedative effects of ethanol through impaired crz production.

## Discussion

High doses of ethanol induce a typical motor impairment and sedation in both mammalian and Drosophila models, similar to the effects of severe ethanol intoxication in humans^18^. The risk for alcoholism is higher for people who show increased resistance to the sedating effects of ethanol, partly because they can consume more alcohol. Thus, although sedation is not a model for alcohol addiction, gaining more insight into the regulation of ethanol responses may be relevant for human studies as well.

Recent work in Drosophila revealed that the transcription factor apontic mediates ethanol-induced sedation via regulating the production of the neuropeptide hormone Crz by a small population of neurosecretory cells in the fly brain^19^. However, the molecular mechanisms that control this response to ethanol still remain poorly understood.

In this work, we identify the core autophagy gene Atg16 as a key component promoting ethanol-induced sedation in Drosophila. Atg16 is a subunit of an E3-like protein complex involved in Atg8a lipidation^9^. Surprisingly, we find that it acts independent of both the E1-like Atg7 and the E2-like Atg3 enzymes that function in the same pathway during autophagy in the larval fat body. Cell type-specific knockdown and genetic rescue experiments reveal that the function of Atg16 also maps to Crz-producing neurosecretory cells, which were previously implicated in the ethanol sedation response in fruit flies^19^.

Importantly, a polymorphism of Atg16 is a genetic risk factor for inflammatory bowel disease^20^,^21^. Atg16 has been suggested to affect the secretory function of Paneth cells, which may influence the intestinal microbiota and sensitize affected human patients and mice to chronic inflammation of the gut^22^. Interestingly, we find that Atg16 regulates Crz expression in Drosophila: both its protein and mRNA levels are reduced in the absence of Atg16 function. The colocalization of Atg16 and Crz within these cells may seem to support a secretory role for Atg16 in this setting, too, but one would expect to see increased rather than decreased level of Crz if there was a secretory defect. It seems more likely that the loss of Atg16 somehow affects crz transcription or mRNA stability. Future studies will be necessary to understand the molecular mechanisms of how Atg16 controls Crz production.

Taken together, we generated and characterized novel Drosophila mutants for Atg16, and showed that in addition to its role autophagy, it also functions in the ethanol-induced sedation response by promoting the production of the neuropeptide Crz in a small group of neurosecretory cells.

## Methods

### Fly stocks and treatments

Flies were maintained at 25 °C, 50% relative humidity and a 12-hour light/12-hour dark daily cycle on media containing 26 g yeast, 66.85 g cornmeal, 0.56 g CaCl_2_, 32.59 g sucrose, 12 g agar, 1.7 ml 0,5% phenylphenol and 10 ml of 25% Nipagin (dissolved in ethanol) per litre of cooked food. P element excisions and fat body cell clones were generated as described earlier^23^. Briefly, we recombined the d129 and d67 mutations onto an FRT82B chromosome and crossed these to virgins hsFlp; QUAS-mCD8-GFP; ET49-QF FRT82B, tub-QS/TM6B, Tb, and the resulting 2- to 4-hour embryos were heat shocked in a 38 °C water bath for 1 hour^23^,^24^. Atg7^d77^ and Atg3^10^ mutants have been previously described^3^,^7^. The Atg3^10^ deletion represents a double knockout for both Atg3 and the neighboring gene Nufip that encodes a RNA binding protein. We generated a transgene that contains Nufip but not Atg3, and used it to construct an Atg3-specific null mutant containing the Atg3^10^ mutation and the rescue transgene for Nufip. This Nufip transgene rescued the larval lethality of Atg3^10^ homozygous mutant animals to pupal semi-lethality with viable adult escapers, but it did not rescue their previously characterized autophagy defect^7^,^13^.

For lifespan analysis, 30 newly eclosed males per genotype were transferred to fresh food every 2–3 days, and the dead flies were counted each time. Negative geotaxis (climbing) assays and paraquat treatments were performed as described^3^,^10^,^25^.

In ethanol sedation tests, 10 males (2 to 5 days old) per genotype were placed into empty vials covered by a piece of silk. The vials of control (w^1119^) and experimental genotypes (all on the w^1119^ genetic background) were placed into the same 1-litre beaker containing a paper towel. 1 ml of 73% ethanol was added onto the paper towel^19^, the beaker was covered with parafilm, and sedated flies were counted in each vial over time. Tub-Gal4 and crz-Gal4 (both obtained from the Bloomington Drosophila Stock Center) was employed to knock down Atg16 using the RNAi line Atg16^KK105993^ (obtained from the Vienna Drosophila Resource Center), and for genetic rescue experiments by expressing UAS-FLAG-Atg16-c on a homozygous Atg16^d67^ mutant background.

### Generation of polyclonal antibodies

Partial Atg16 coding sequences were amplified using primers ATGTCTACGGAGGAGCATGTGTG and ATCTTATTAGGGCTCACGAACCGCGTC (for recombinant Atg16 domain expression), or GGATCCGCATATGAGAAAAAGACTGCCGTCAATTTTCA and GTCGACTCGAGTTAATTTTTGCCAATGTCCCAAAGTT (for recombinant Atg16 linker region expression). The PCR products were phosphorylated, blunt cloned into XmnI–EcoRV-digested dephosphorylated pENTR1A (Invitrogen), and subsequently recombined into pDEST17 (Invitrogen). N-terminally His-tagged protein was expressed in the *Escherichia coli* Rosetta strain (EMD Millipore) and purified using Ni-agarose beads (Qiagen/Biomarker). Recombinant proteins were used to immunize rats in-house following standard procedures utilizing Freund’s adjuvants (Sigma).

### Generation of transgenic lines

For the generation of genomic promoter-driven Atg16-HA rescue transgene, we PCR amplified a 8,206 bp region containing the Atg16 gene^11^ and its promoter using primers AATGCGGCCGCGGCGCGCCGTCTCCAGCGACATGGTCAGCA and TATTGCGGCCGCATGGCGCGCCTGACTCCGAGTAGATGGTGCAGCG, and cloned it into pGen-3xHA vector^23^ as an AscI fragment. We followed the same strategy to generate a genomic promoter-driven Crz-mCherry transgene, by amplifying a 1,561 bp genomic region of the crz gene^11^ using primers TACTAGGCGCGCCAGATCTTGCTACTTCCTGCTGCGGCG and CTCGAGGCGCGCCTGAATTCATGTTTTCCAAACACATTGGGTTCGGC, and cloning it into our BglII-EcoRI digested pGen-3xmCherry vector. In these constructs, the stop codons were replaced by 3xHA or 3xmCherry coding sequences, respectively. The untagged Nufip rescue transgene was generated by amplifying a 4,042 bp genomic region^11^ using primers GCTAGCGGCCGCCAGTGTCAATCCCACTTTGTGGAC and GCTAGCGGCCGCGTGGAAAATCGCAGCACATACCCTT, and then cloning the resulting PCR product into pCasper5 using NotI. UAS-FLAG-Atg16-c was generated by cloning a full length Atg16 isoform c coding sequence (PCR amplified from cDNA using primers TATAGCGGCCGCGGGAATGTCTACGGAGGAGCATGTG and CTCGAGGTACCTATGACTCCGAGTAGATGGTGC) into NotI-Acc65I digested UAS-3xFLAG vector^25^. Transgenic lines were established by Bestgene. Two independent insertions of UAS-Atg16-c were tested with similar results, and one of these is shown in figures.

### Quantitative real-time PCR (qPCR)

RNA samples were prepared from 20 mg of wandering larvae for Atg16 expression analysis, and from adult flies treated with alcohol vapor for 30 min for crz expression analysis, using Direct-zol RNA MiniPrep Kit (Zymo Res), followed by cDNA synthesis using High-Capacity cDNA Reverse Transcription Kit (Applied Biosystems) according to the manufacturer’s instructions. PCR reactions were performed on a Rotor-Gene Q instrument (QIAGEN) with gene-specific primers and Rotor-Gene SYBR Green Kit (QIAGEN). The primer sequences are AAAAGCTTACAAAATGTGTGACGA and CAATCGATGGGAAGACGG for Actin5C; AGGCCCACGAGAACGAGT and GGTGGAATTTTTGCCAATGT for Atg16; and GGCTCGAGCGCTGTCTATC and ACTCGGTTGGCATTGAAGTC for crz. Relative expression ratios were calculated by normalizing to Actin5C.

### Histology and imaging

For starvation treatments, L3 stage larvae were floated in 20% sucrose for 3 hours. LTR staining was performed on the fat body of dissected larvae by incubating in 100 nM Lysotracker Red (Invitrogen) for 5 min. For immunofluorescent labeling, brains of male flies that were treated with alcohol vapor for 30 min were dissected and fixed in 4% paraformaldehyde at room temperature for 30 min, blocked in PBS with 0.1% Triton X-100 and 10% normal goat serum (PBSTG) for 4 hours, incubated in primary antibody/PBSTG solution for 2 consecutive nights and in secondary antibody/PBSTG for overnight at 4 °C.

Images were taken on a grid confocal microscope (Zeiss Axioimager M2 with Apotome2), except for the analysis of Crz production, for which laser scanning confocal microscopes (Olympus Fluoview FV1000 and Leica SP5 AOBS) were used with identical settings for all genotypes. Primary images were adjusted in Photoshop (Adobe) to produce final figures. Tissues were processed for transmission electron microscopy as described previously^3^,^25^.

### Western blots and antibodies

Samples were processed for western blots as described^23^,^25^. The following primary antibodies were used: rat anti-Atg16/1 (WB 1:5,000; this study), rat anti-Atg16/2 (WB 1:5,000; this study), rabbit anti-Atg8 (WB 1:5,000)^24^, rabbit anti-p62 (IF 1:2,000, WB 1:5,000)^8^, mouse anti-Tubulin (WB 1:2,000; DSHB AA4.3s), rat anti-HA (IF 1:150, Roche), rabbit anti-Crz (IF 1:500, a gift from Jan Veenstra)^26^. Alexa 488 anti-rabbit, Alexa 488 anti-rat, Alexa 568 anti-rabbit, Alexa 568 anti-rat secondary antibodies were used for IF (1:1,500, Invitrogen), and alkaline phosphatase–conjugated anti-rat, anti-rabbit, anti-mouse secondary antibodies were used for WB (1:5,000; Millipore).

### Statistics

Sedation time 50 data were imported into SPSS Statistics version 20 (IBM) and visualized with the Scatter/Dot graph. After checking for normality of data distribution in SPSS, we used one-way ANOVA to evaluate climbing assays and Kaplan-Meier log-rank test for lifespan and paraquat experiments. In the case of alcohol sedation experiments, data on control and mutant genotypes were compared from the same experiment in which 2–4 vials (each containing flies of different genotypes) were placed in the same beaker, and we used two-tailed, two-sample paired t tests for comparing 2 genotypes or two-way ANOVA for comparing 3 or 4 genotypes. Two-tailed, two-sample, unpaired t tests were used to evaluate Crz expression data. Pearson correlation coefficients, cell fluorescence intensity calculated from area, integrated density and mean gray values were all obtained using ImageJ (NIH).

## Additional Information

**How to cite this article**: Varga, K. *et al*. Loss of Atg16 delays the alcohol-induced sedation response via regulation of Corazonin neuropeptide production in Drosophila. *Sci. Rep.* **6**, 34641; doi: 10.1038/srep34641 (2016).
